# Unveiling Berberine analogues as potential inhibitors of *Escherichia coli* FtsZ through machine learning molecular docking and molecular dynamics approach

**DOI:** 10.1038/s41598-025-98835-x

**Published:** 2025-04-26

**Authors:** Aditi Roy, Anand Anbarasu

**Affiliations:** 1 Medical and Biological Computing Laboratory, School of Bio-Sciences and Technology (SBST), Vellore Institute of Technology (VIT), Vellore, Tamil Nadu 632014 India; 2 Department of Biotechnology, School of Bio-Sciences and Technology (SBST), Vellore Institute of Technology (VIT), Vellore, Tamil Nadu 632014 India

**Keywords:** Antimicrobial resistance, *Escherichia coli*, FtsZ, Molecular docking, Molecular dynamics simulation, Machine learning, Biotechnology, Computational biology and bioinformatics, Drug discovery, Microbiology, Structural biology

## Abstract

The bacterial cell division protein FtsZ, a crucial GTPase, plays a vital role in the formation of the contractile Z-ring, which is essential for bacterial cytokinesis. Consequently, inhibiting FtsZ could prevent the formation of proto-filaments and interfere with the cell division machinery. The remarkable conservation of FtsZ across diverse bacterial species makes it a promising drug target for combating drug resistance. In the present study, 1072 berberine analogues were screened for favorable pharmacokinetic properties. A total of 60 compounds that fulfilled the drug-likeliness criteria and were found to be non-toxic were selected for virtual screening against *Escherichia coli* FtsZ protein (PDB ID: 8GZY). Molecular docking revealed a strong binding affinity of ZINC000524729297 (− 8.73 kcal/mol) and ZINC000604405393 (and − 8.55 kcal/mol) with FtsZ by strong intermolecular hydrogen bonds and hydrophobic interactions. Subsequently, the docking profiles were validated through a 500 ns MD simulation and MMPBSA analysis of the FtsZ-ligand complexes. The analysis revealed the FtsZ- ZINC524729297 and FtsZ-ZINC000604405393 complexes had the lowest root-mean-square deviation with lowest binding energy and enhanced conformational stability in a dynamic environment. These findings suggest that ZINC524729297 and ZINC000604405393 are the potent lead compound that targets FtsZ and requires further experimental validation.

**Supplementary Information:**

The online version contains supplementary material available at 10.1038/s41598-025-98835-x.

## Introduction

*Escherichia coli* (*E. coli*) is a Gram-negative, rod-shaped, facultative anaerobic bacterium frequently isolated from adult patients with bacteremia^1^ and can be life-threatening in severe cases^2^. In recent years, the rate of bacteremia has increased steadily^3^. In general, most *E. coli* strains colonize the human gastrointestinal tract as normal flora^4^. While most strains are harmless, some can cause intestinal or extraintestinal infections due to specific virulence factors (VFs)^5^. Strains that can access and survive in the bloodstream are known as extraintestinal pathogenic *E. coli* (ExPEC) and are associated with a range of infections including urinary tract infections (UTIs), septicemia, and meningitis^6^. These bacteria most frequently colonize the urinary tract and often become a primary source of infections in the bloodstream^7^, resulting in approximately 120,000–242,000 deaths annually. Bacterial infections can be fatal and significantly increase mortality rates among affected people. The discovery of antibiotics, often referred to as “miracle drugs,” has drastically reduced patient mortality rates^8^. However, excessive and improper use of these drugs has resulted in the emergence of antimicrobial resistance (AMR), leading to a major global health challenge^9^. Recent reports indicate that AMR is linked to approximately 5 million deaths in 2019, which is more than twice the number attributed to COVID-19 in 2020. According to Jim O’Neil, AMR is projected to cause 300 million deaths annually by 2050, with an estimated global economic impact of up to $100 trillion^10^. Previous studies have shown that *E. coli* exhibits nearly 70% resistance against streptomycin and sulfisoxazole-tetracycline. Other studies have also reported the reduced susceptibility of *E. coli* to other β-lactam antibiotics and aminoglycoside drugs^11^. Exploring new compounds that selectively bind to key proteins involved in the bacterial cell cycle rather than penicillin-binding proteins holds promise for the development of new classes of antibacterial agents. Bacterial cell division is a dynamic biological process involving numerous essential proteins, making them potential targets for the development of antibacterial drugs^12^. In Gram-negative bacteria, cell division is regulated by 12 proteins, among which filamentous temperature-sensitive mutant Z (FtsZ) is an essential cytokinesis protein, that is highly conserved in all bacteria^13^. It has a crucial role in prokaryotic cell division. This bacterial cell division process is a new and attractive target for developing new antibacterial drug^14^. Interrupting the function of this protein can helps to overcome drug resistance^15^. FtsZ is homologous to eukaryotic tubulin, but exhibits very little sequence identity with it, suggesting that drugs targeting FtsZ will be less toxic to eukaryotic cells^16^. The protein assembles into a filamentous structure resembling tubulin by arranging its head and tail. This results in the formation of a ring-like structure known as the Z-ring at the centre of the cell during cytokinesis. Once the Z-ring is formed through GTP-dependent self-polymerization, essential proteins such as FtsA, FtsI, and ZipA are recruited at the cell division site to initiate new cell wall synthesis^17^. FtsZ consists of N-terminal and C-terminal globular domains, separated by a core H7 helix and a spacer T7 loop. The N-terminal domain is responsible for GTP binding which is the natural substrate, while the C-terminal domain is involved in GTP hydrolysis. Two notable drug-binding sites present in FtsZ are the nucleotide-binding domain (NBD) and inter-domain cleft (IDC)^18^. The NBD shares more similarity with tubulin, as the glycine-rich motif GGGTG(T/S)G is present in both proteins^19^. Therefore, drugs targeting the FtsZ GTP-binding site may interact with tubulin, potentially causing toxicity in mammalian cells. In contrast, IDC exhibits less similarity to tubulin, reducing the likelihood of toxicity in mammalian cells^18^. The T7 loop and the C-terminal domain present in IDC are highly conserved both at the sequence and structure levels, and this loop of one subunit is intercalated into the NBD of another subunit and initiates GTP hydrolysis^20^. Therefore, blocking and targeting the IDC of FtsZ could inhibit GTPase activity and inhibit cell division. Berberine is a plant derived alkaloid with medicinal properties and used in China and native America^21^. It exhibits numerous biological activities, including antimicrobial, antifungal, and antiviral properties, and is involved in the inhibition of cell adhesion and migration^22^. Studies have shown that berberine contains three aromatic rings and one nitrogen atom that can inhibit the FtsZ protein in some bacterial species^23^. Evidence indicates that berberine has anti-diabetic activity by activating AMPK by increasing the AMP/ATP ratio in the mitochondria^24^. Additionally, berberine showed activity against Gram-positive bacteria with minimum inhibitory concentration (MIC) values ranging from 100 to 400 µg/mL by targeting FtsZ^25^. Furthermore, berberine has been reported to inhibit *E. coli* FtsZ^26^; however, it is necessary to develop new compounds with specific targets to prevent infection caused by *E. coli*.

**Fig. 1:**
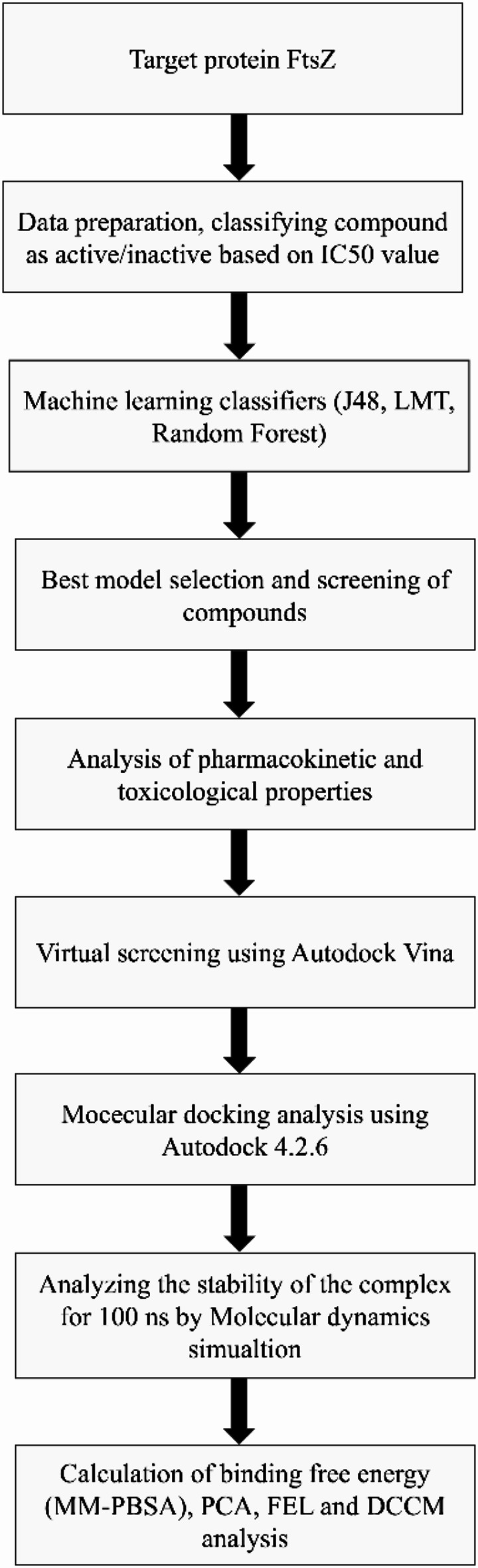
Flowchart depicting the virtual screening process for identifying effective FtsZ inhibitors.

In this study, we explored the effectiveness of berberine as an inhibitor of the FtsZ protein in *E. coli*. Here, 1072 berberine analogues were screened based on ADMET properties and molecular docking to discover new compounds, followed by molecular dynamics simulations and other analyses to validate the results as shown in Fig. 1.

## Results

### Performance of different machine-learning (ML) classifiers and data set training

**Fig. 2:**
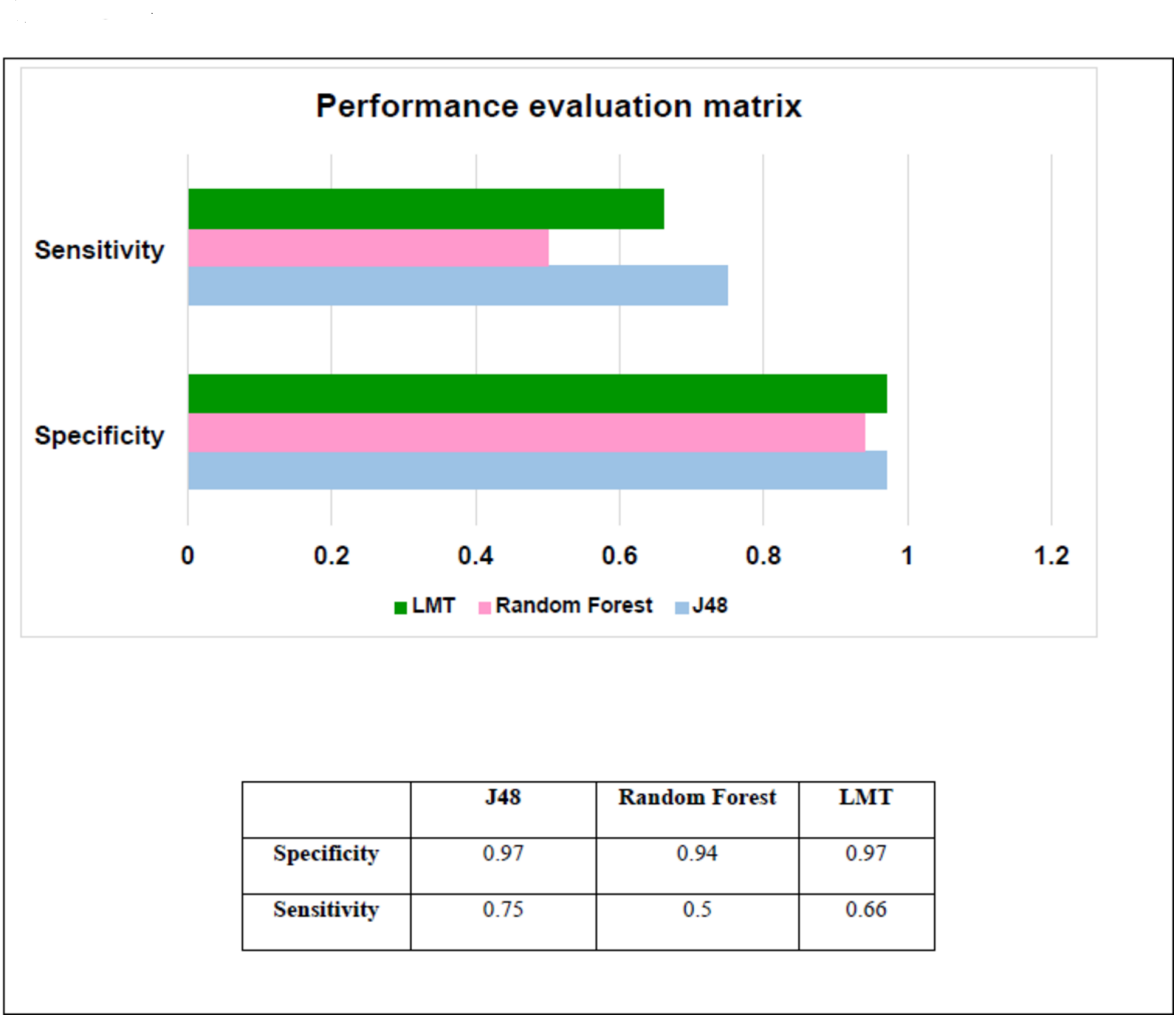
Statistical performance of various classifiers for development of machine learning model in the training dataset.

To develop an optimal ML model for differentiating anti-FtsZ compounds from non-inhibitory compounds, we built classification model using ML techniques. The performance of the model was evaluated using several statistical metrics, as given in Table 1. Three different classification algorithms, namely random forest, LMT, and J48, were used to train a set of models on the training dataset using ten-fold cross-validation (CV). The kappa statistic values represent the consistency between the actual and predicted model classes during model evaluation, and a value of 1 represents perfect agreement between the ground truth and the classification of the classifier model. The J48 model showed the highest kappa statistic value of 0.62 with a final root mean square error (RMSE) value of 0.23. The analysis showed that J48 was the best classifier overall, followed by LMT and random forest. The accuracy rates of the three models (J48, LMT, and random) were 93.18%, 90.90%, and 88.63%, respectively. The best models for each dataset were identified by sensitivity and specificity plot to evaluate the classifier’s accuracy in correctly recognizing positively and negatively labeled instances, as shown in Fig. 2. The sensitivity and specificity ranged from 50 to 75% and 94–97% respectively. J48 was the most sensitive classifier for the dataset, and random forest was the least sensitive classifier. Furthermore, other performance metrices, such as receiver operating characteristics (ROC) curve analysis, were used to validate the robustness of the model. The performance of the binary classifier model was shown by the ROC curve during the change in the discrimination threshold. The ROC curve of the current model initially showed a very strong association with the true positive rate axis, indicating maximizing the true positive rate while minimizing false positive rates (maximizing specificity and sensitivity). The ROC of J48, random forest and LMT were 0.95, 0.87 and 0.79 respectively. J48 was selected as the best model based on its performance and selected for screening of compounds. Out of 1072 berberine analouges 740 compounds were predicted to be active by the model and were considered for further evaluation.

**Table 1:** Comparison of performance of various classifiers to develop screening model in the training dataset.

Classifier name	Correctly classified instances % (value)	Kappa statistic	Mean absolute error	Root mean square error	MCC	ROC
J48	93.18	0.62	0.06	0.23	0.634	0.951
Random forest	88.63	0.38	0.13	0.28	0.385	0.874
LMT	90.9	0.45	0.16	0.3	0.471	0.795

### ADMET analysis and virtual screening

We have retrieved the structure of 740 berberine analogues from the PubChem database due to their reported activity against FtsZ protein for ligand-based virtual screening (Supplementary Information SI1). A total of 571 lead compounds were screened and selected based on pharmacokinetic properties, such as molecular weight, hydrogen bond donor and acceptor, total polar surface area, high GI absorption, no violations of Lipinski’s rule, and low synthetic accessibility using SwissADME (Supplementary Information SI2). Furthermore, these compounds were evaluated for their hepatotoxicity, cardiotoxicity, carcinogenicity, and mutagenicity. After toxicity screening, 60 compounds were found to be non-toxic (Supplementary Information SI3). The selected compounds were further screened by calculating their binding affinities using PyRx (Autodock Vina) (Supplementary Information SI4). Finally, 20 compounds with binding affinity < − 7.4 Kcal/mol were docked to evaluate their binding energies and inhibition constant (IC) using Autodock 4.2.6.

### Molecular docking analysis of protein–ligand complex

**Fig. 3:**
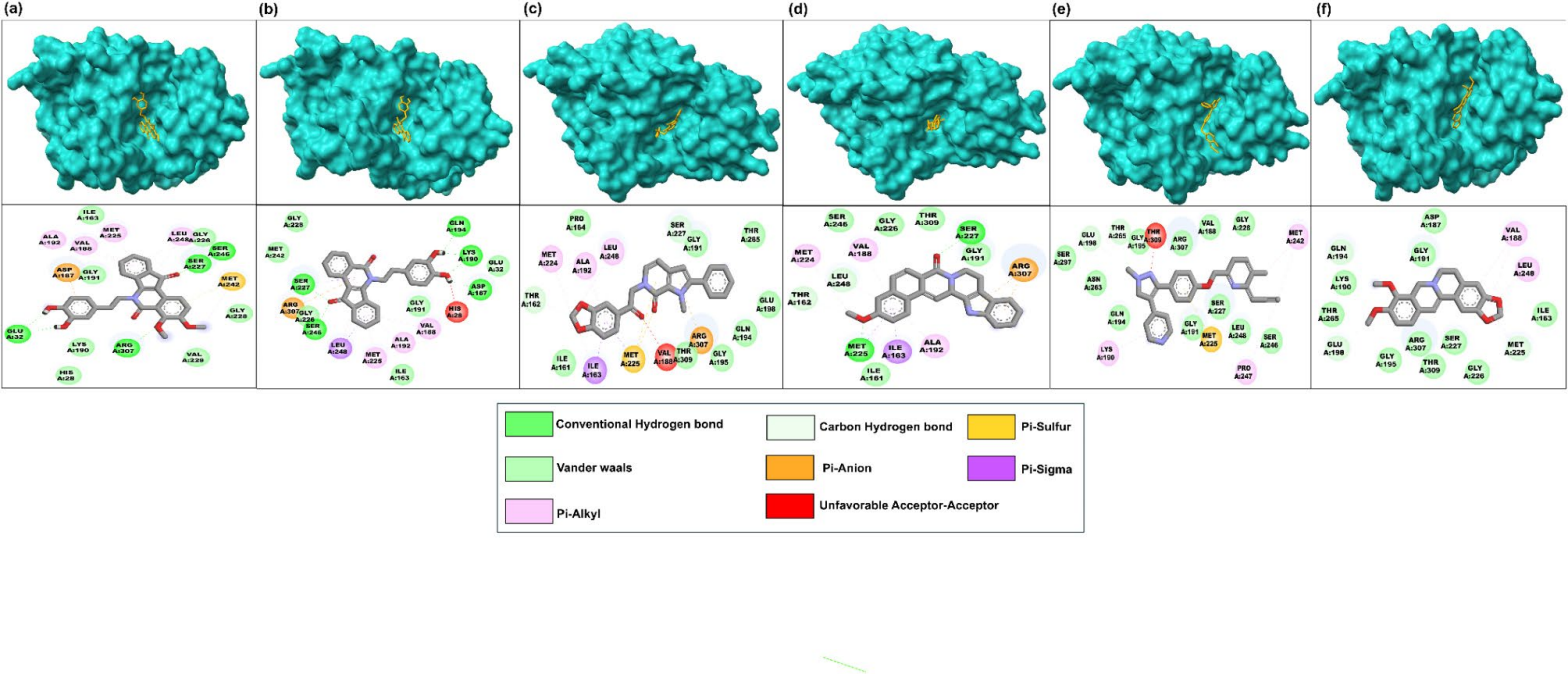
Molecular docking interaction of ligands with FtsZ (**a**) FtsZ-ZINC524729297, (**b**) FtsZ-ZINC000604405393, (**c**) FtsZ-ZINC000072312902, (**d**) FtsZ-ZINC000085341281, (**e**) FtsZ-DB08387, (**f**) FtsZ-Berberine.

In this study, intermolecular site-specific docking was performed in triplicates to analyze the binding interactions between FtsZ and the berberine analogues (Supplementary Information SI5). This offers an understanding of the molecular mechanism underlying the activity of the compound at the atomic level. Our findings suggest that molecular docking of FtsZ with five compounds, ZINC000524729297, ZINC000604405393, ZINC000072312902, ZINC000085341281 and DB08387 resulted in a high binding affinity complex. The docking of compound ZINC524729297 resulted in a complex with a binding energy of − 8.73 kcal/mol and an IC value of 0.396 µM. Other compounds like ZINC000604405393, ZINC000072312902, ZINC000085341281 and DB08387 showed a binding energy of − 8.55 kcal/mol, − 8.32 kcal/mol, − 8.3 kcal/mol, − 8.2 kcal/mol, and IC 0.543 µM, 0.799 µM, 0.816 µM, 0.971 µM, respectively. The binding energy of the reference compound berberine with FtsZ showed binding energy of − 6.59 kcal/mol and IC of 14.79 µM. We analyzed various types of intermolecular interactions, including hydrogen bonds, van der Waals forces, Pi-alkyl, Pi-sigma, and Pi-Sulfur, which were involved in the active site of the FtsZ protein and the inhibitors (Table 2). Molecular docking interactions of the docked complexes are depicted in Fig. 3a–f. The inhibitor ZINC524729297 interacts with FtsZ through four hydrogen bonds with the residues Glu 32, Ser 227, Ser 246, Arg 307 and seven van der Waals interactions with His 28, Ile 163, Lys 190, Gly191, Gly 226, Gly 228, and Val 229, and other interactions with Asp 187, Val 188, Ala 192, Met 225, Met 242, and Leu 248 (Fig. 3a). The ZINC000604405393-FtsZ complex was stabilized by four hydrogen bonds with residues Asp187,Lys190, Gln194, Ser246; van der Waals interactions, Pi-cation, Pi-sigma and Pi-alkyl with residues, Glu32, Ile163, Gly191, Gly226, Gly,228, Met242, Arg307, Leu248, Val188, Ala192 and Met225, repectively (Fig. 3b). Another complex FtsZ- ZINC000072312902 had van der Waals interactions with the residues Ile161, Pro164, Gly191, Gln194, Gly195, Glu198, Thr 265 and Thr 309; other interactions like Pi-sigma Pi-sulfur and Pi-alkyl with Ile163, Met225 and Ala192, Met224 and Leu248 (Fig. 3c). FtsZ- ZINC000085341281 showed two hydrogen bonds with Met225, Ser227 and five van der Waals interactions with Ile161, Gly191, Gly226, Ser246 and Thr309 residues shown in Fig. 3d. The FtsZ- DB08387 complex exhibited eleven van der Waals interactions, three Pi-alkyl and one Pi-sulfur with the residues Val188, Gly191, Gln194, Gly195, Ser227, Gly228, Ser246, Leu248, Asn263, Ser297, Arg307, Lys190, Met242, Pro247 and Met 225, respectively (Fig. 3e). The berberine-FtsZ complex showed eleven van der Waals interactions, and two Pi-alkyl interactions with Ile163, Asp187, Lys190, Gly191, Gly195, Gly226, Ser227, Arg307, Thr309, Thr265, Val188 and Leu248, respectively. (Fig. 3f).

**Table 2:** Different molecular interactions of screened compounds.

Ligands	Structure	Binding energy (Kcal/mol)	Inhibition constant (µM)	Hydrogen bonds	Other interactions
ZINC000524729297		− 8.73	0.396	Glu32, Ser227, Ser246, Arg307	His28, Ile163, Asp187, Val188, Lys190, Gly191, Ala192, Met225, Gly226, Gly228, Val229, Met242, Leu248
ZINC000604405393		− 8.55	0.543	Asp187,Lys190, Gln194, Ser246	His28, Glu32, Ile163, Gly191, Val188, Ala192, Met225, Gly226, Gly228, Met242, Leu248, Arg307
ZINC000072312902		− 8.32	0.799	NA	Ile161, Thr162, Ile163, Pro164, Val188, Gly191, Ala192, Gln194, Gly195, Glu198, Met224, Met225, Ser227, Leu248, Thr265, Arg307, Thr309
ZINC000085341281		− 8.3	0.816	Met225, Ser227	Ile161, Thr162, Ile163, Val188, Gly191, Ala192, Met224, Gly226, Ser246, Leu248, Arg307, Thr309
DB08387		− 8.2	0.971	NA	Val188, Lys190, Gly191, Gln194, Gly195, Glu198, Met225, Ser227, Gly228, Met242, Ser246, Pro247, Leu248, Asn263, Thr265, Ser297, Arg307, Thr309
Berberine		− 6.59	14.79	NA	Ile163, Asp187, Val188, Lys190, Gly191, Gln194, Gly195, Glu198, Met225, Gly226, Ser227, Arg307, Thr309, Leu248, Thr265

### Molecular dynamic simulations

**Fig. 4:**
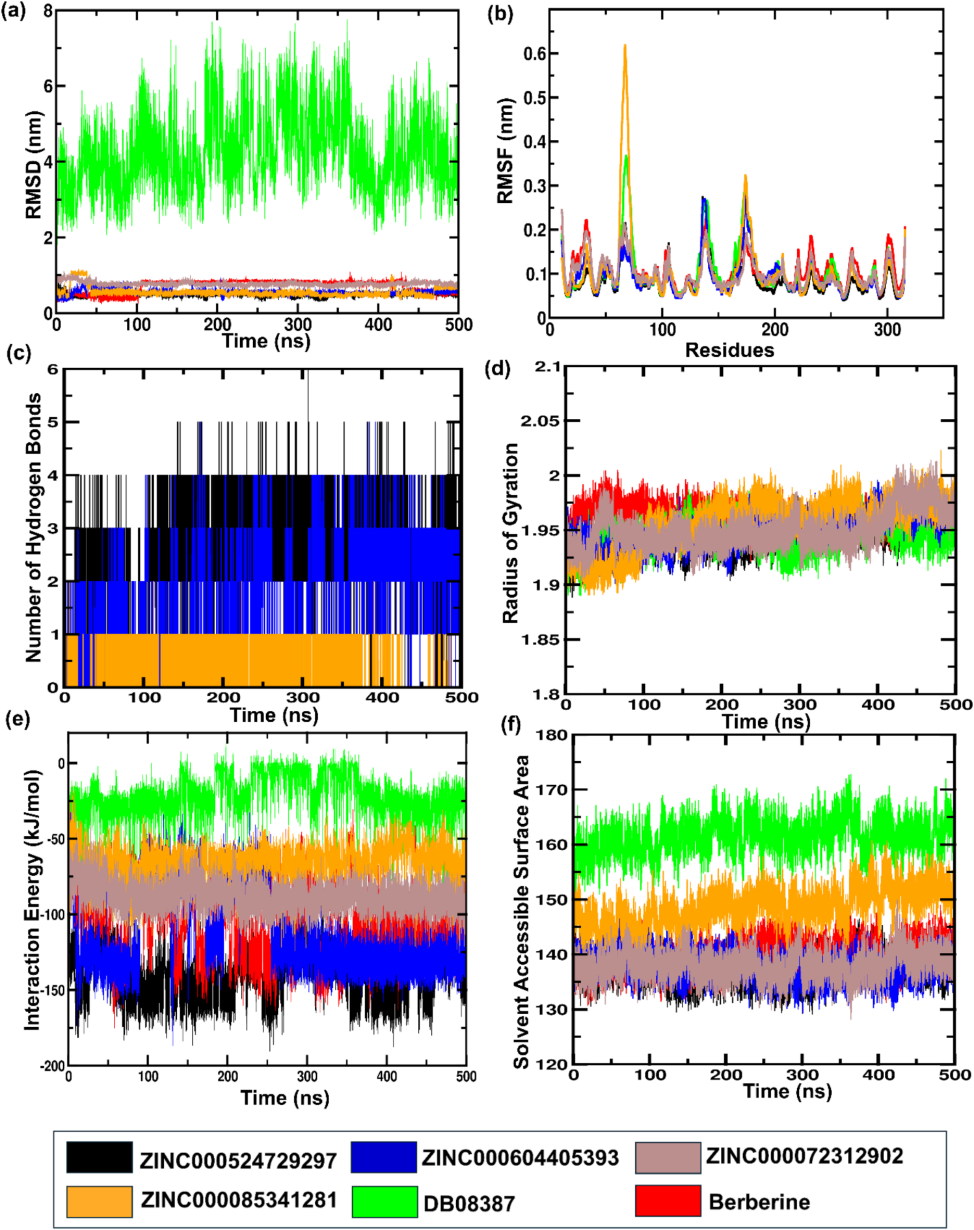
Molecular dynamics simulation analysis of protein–ligand (**a**) Root means square deviation (RMSD), (**b**) Root mean square fluctuation (RMSF) associated with the number of residue, (**c**) Intermolecular H-bond, (**d**) Radius of gyration, (**e**) Interaction energy, (**f**) Solvent Accessible Surface Area (SASA).

The protein and inhibitor complexes were further subjected to MD simulation to evaluate the movement of atoms of the entire macromolecule over a specified time step of 500 ns. The root-mean-square deviation (RMSD) measures the deviation in atomic positions within the amino acid structure during ligand binding. A lower RMSD value indicated greater stability of the complex, reflecting minimal deviations. The RMSD values for the complexes of ZINC000524729297, ZINC000604405393, ZINC000072312902, ZINC000085341281 and DB08387 with FtsZ were 0.475 nm, 0.551 nm, 0.775 nm, 0.550 nm and 4.21 nm, respectively, while berberine-FtsZ showed an RMSD of 0.707 nm (Fig. 4a). The root-mean-square fluctuation (RMSF) is used to represent the fluctuation of the amino acid residues in the protein while interacting with the ligand. The RMSF values revealed that ZINC000524729297, ZINC000604405393, ZINC000072312902, ZINC000085341281, DB08387 and berberine had fluctuations of 0.085 nm, 0.093 nm, 0.099 nm, 0.103 nm, 0.103 nm and 0.107 nm respectively (Fig. 4b). The interaction between FtsZ-ZINC524729297, FtsZ-ZINC000604405393 and FtsZ-ZINC000085341281 showed consistent hydrogen bonding with an average H-bond of 2.3, 1.2 and 0.17, respectively, while other complexes showed no hydrogen bond, thereby conferring enhanced stability to the ZINC524729297 protein complex (Fig. 4c). The compactness of the complexes FtsZ-ZINC524729297, FtsZ-ZINC000604405393, FtsZ-ZINC000072312902, FtsZ-ZINC000085341281, FtsZ-DB08387 and FtsZ-berberine were analyzed through radius of gyration (Rg), which showed an average value of 1.94 nm, 1.95 nm, 1.94 nm, 1.95 nm, 1.94 nm, and 1.96 nm, respectively (Fig. 4d). The interaction energy was calculated for the protein–ligand complex, as shown in Fig. 4e. The average interaction energy of FtsZ-ZINC524729297 was − 140.168 kcal/mol, FtsZ- ZINC000604405393 was − 112.89 kcal/mol, which are lower than FtsZ-berberine complex (− 105.64 kcal/mol). The other complexes like FtsZ- ZINC000072312902, FtsZ-ZINC000085341281 and FtsZ-DB08387 exhibited higher average interaction energy of − 88.17 kcal/mol, − 65.33 kcal/mol and − 21.197 kcal/mol, respectively. The solvent-accessible surface area (SASA) was also measured for ZINC524729297 (137.43 nm^2^), ZINC000604405393, (138.10 nm^2^), ZINC000072312902 (138.02 nm^2^), ZINC000085341281 (148.69 nm^2^), DB08387 (161.49 nm^2^) and berberine and berberine (139.93 nm^2^), as shown in Fig. 4f. We have performed 500ns simulation in triplicates for these two best complexes FtsZ-ZINC524729297 and FtsZ- ZINC000604405393 given in Supplementary Information SI6.

### Binding free energy calculation

**Fig. 5:**
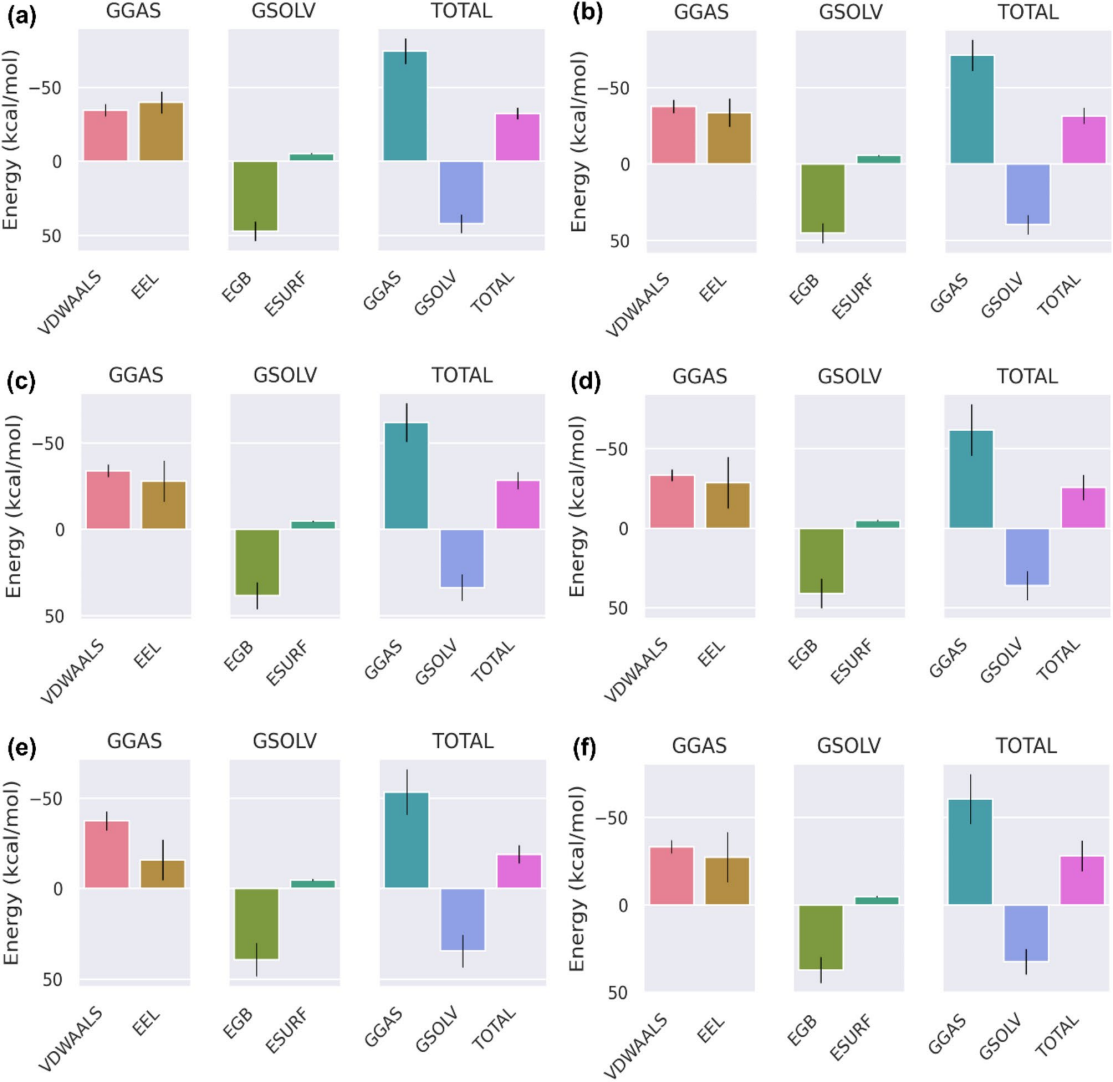
MM-PBSA analysis of individual residues contributing to total binding free energy (**a**) FtsZ-ZINC524729297, (**b**) FtsZ-ZINC000604405393, (**c**) FtsZ-ZINC000072312902, (**d**) FtsZ-ZINC000085341281, (**e**) FtsZ-DB08387, (**f**) FtsZ-Berberine.

The MM-PBSA analysis was performed for a better understanding of the molecular interactions between FtsZ and ligand molecules. The binding energy was determined for a specific time of 500 ns using the MD simulations given in Table 3. The analysis provided various energy calculations, including van der Waals molecular mechanics energy (VDWAALS), electrostatic energy (EEL), polar solvation energy (EGB), solvent-accessible surface area energy (ESURF), total gas phase energy (GGAS), total solvation energy (GSOLV), and the total binding energy (GTOTAL). The total binding energies of the complexes FtsZ-ZINC524729297, FtsZ-ZINC000604405393, FtsZ- ZINC000072312902, FtsZ-berberine, FtsZ-ZINC000085341281 and FtsZ-DB08387 were − 32.48 ± 3.84 kcal/mol, − 31.58 ± 5.26 kcal/mol, − 28.38 ± 5.02 kcal/mol, − 28.14 ± 8.72 kcal/mol, − 25.71 ± 8.02 kcal/mol and − 19.02 ± 5.04 kcal/mol respectively are presented in Fig. 5. The FtsZ-ZINC524729297 and FtsZ-ZINC000604405393 complexes exhibited the lowest total binding energy, indicating strong interactions between the protein and ligand compared to berberine and other ligands.

**Table 3:** Binding free energy calculation by MM/PBSA analysis.

Compounds	VDWAALS (kcal/mol)	EEL (kcal/mol)	EGB (kcal/mol)	ESURF (kcal/mol)	GGAS (kcal/mol)	GSOLV (kcal/ mol)	TOTAL (kcal/mol)
ZINC524729297	− 34.63 ± 4.13	− 39.87 ± 7.41	47.21 ± 6.55	− 5.18 ± 0.42	− 74.51 ± 8.7	42.03 ± 6.26	− 32.48 ± 3.84
ZINC000604405393	− 37.69 ± 4.34	− 33.58 ± 9.25	45.26 ± 6.63	− 5.57 ± 0.5	− 71.27 ± 10.17	39.69 ± 6.4	− 31.58 ± 5.26
ZINC000072312902	− 34.11 ± 3.62	− 27.97 ± 11.95	38.48 ± 7.84	− 4.78 ± 0.33	− 62.07 ± 11.2	33.7 ± 7.75	− 28.38 ± 5.02
ZINC000085341281	− 33.29 ± 3.61	− 28.61 ± 16.18	41.03 ± 9.36	− 4.84 ± 0.44	− 61.9 ± 16.27	36.19 ± 9.19	− 25.71 ± 8.02
DB08387	− 37.49 ± 5.22	− 16.02 ± 11.2	39.25 ± 9.28	− 4.77 ± 0.61	− 53.51 ± 12.63	34.48 ± 9.01	− 19.02 ± 5.04
Berberine	− 33.25 ± 3.76	− 27.4 ± 14.36	37.29 ± 7.46	− 4.77 ± 0.46	− 60.65 ± 14.25	32.52 ± 7.26	− 28.14 ± 8.72

### Principal component analysis (PCA)

**Fig. 6:**
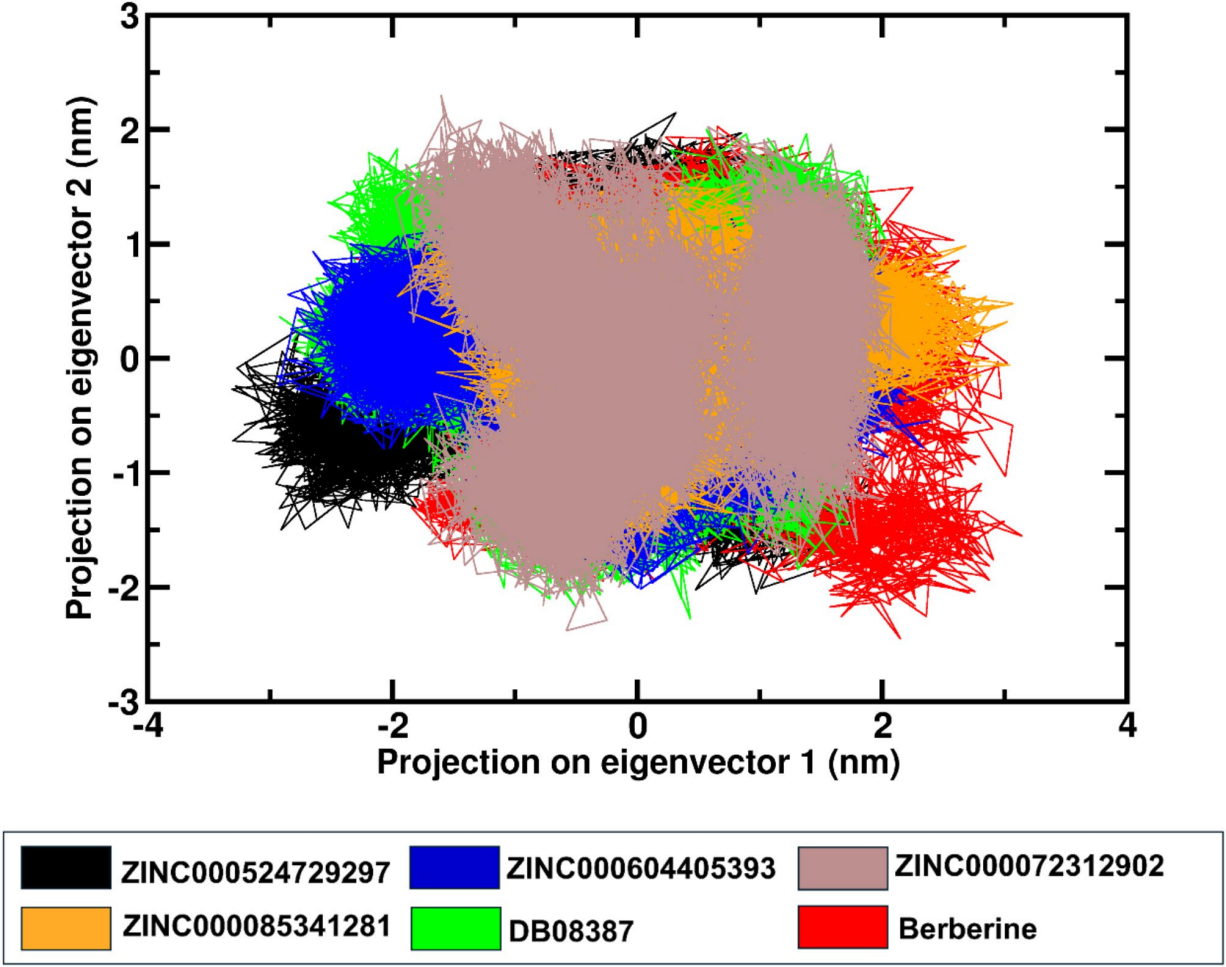
The 2D conformational projection of Principal Component Analysis (PCA) of FtsZ-ZINC524729297, FtsZ-ZINC000604405393, FtsZ-ZINC000072312902, FtsZ-ZINC000085341281, FtsZ-DB08387, FtsZ-Berberine.

PCA analysis was performed to gain a better understanding of the impact of inhibitor binding on FtsZ’s motion and to analyze specific structural alterations due to atomic movements. The coordinate motions in the 500 ns MD simulation time were recorded in the covariance matrix, and eigenvectors were formed to represent the trajectory motion. The role of ligand binding in FtsZ motion was represented by a two-dimensional (2D) projection plot to compare the dynamicity of the protein complexes. When FtsZ binds to specific ligands to form complexes, the shared conformational space increases the likelihood of stability as depicted in Fig. 6.

### Free energy landscape (FEL)

**Fig. 7:**
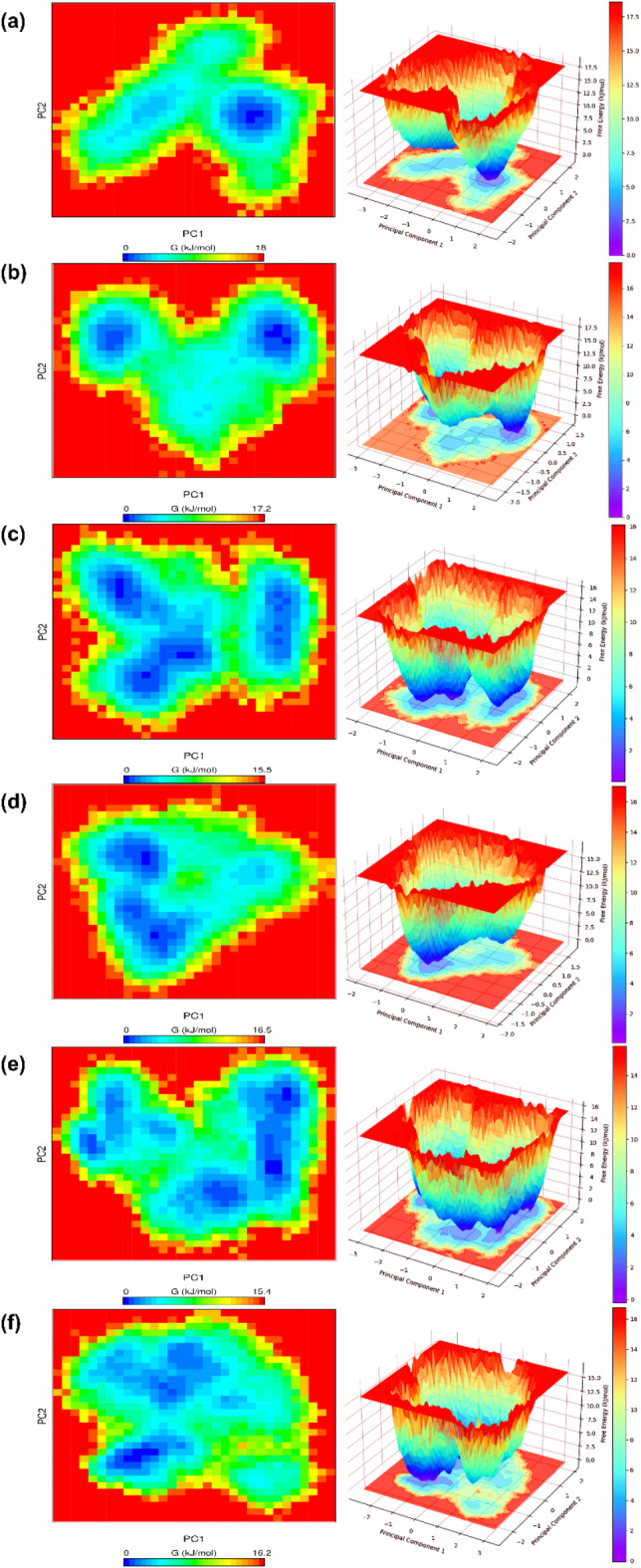
The graphical depiction of PCA based Free Energy Landscape (FEL) analysis of FtsZ-ligand complexes (**a**) FtsZ-ZINC524729297, (**b**) FtsZ-ZINC000604405393, (**c**) FtsZ-ZINC000072312902, (**d**) FtsZ-ZINC000085341281, (**e**) FtsZ-DB08387, (**f**) FtsZ-Berberine.

The FEL analysis helps to understand conformational changes in proteins based on energy and time. The first two principal components (PC1 and PC2) were used to analyze Gibbs free energy. The blue area in the free energy map represents the lower energy with a more stable state, whereas the red zone corresponds to higher energy with a less stable conformation. We calculated the Gibbs free energy values for all the complexes and the finding reveals that the free energy landscape values for FtsZ-ZINC524729297 (18 kJ/mol), FtsZ-ZINC000604405393 (17.2 kJ/mol), FtsZ-ZINC000072312902 (15.5 kJ/mol), FtsZ-ZINC000085341281 (16.5 kJ/mol), FtsZ-DB08387 (15.4 kJ/mol) and FtsZ-Berberine (16.2 kJ/mol) respectively as shown in Fig. 7a–f. These results suggest that the FtsZ- ZINC524729297 and FtsZ-ZINC000604405393 complexes were thermodynamically more stable than FtsZ-berberine.

### Dynamic cross-correlation matrix (DCCM)

**Fig. 8:**
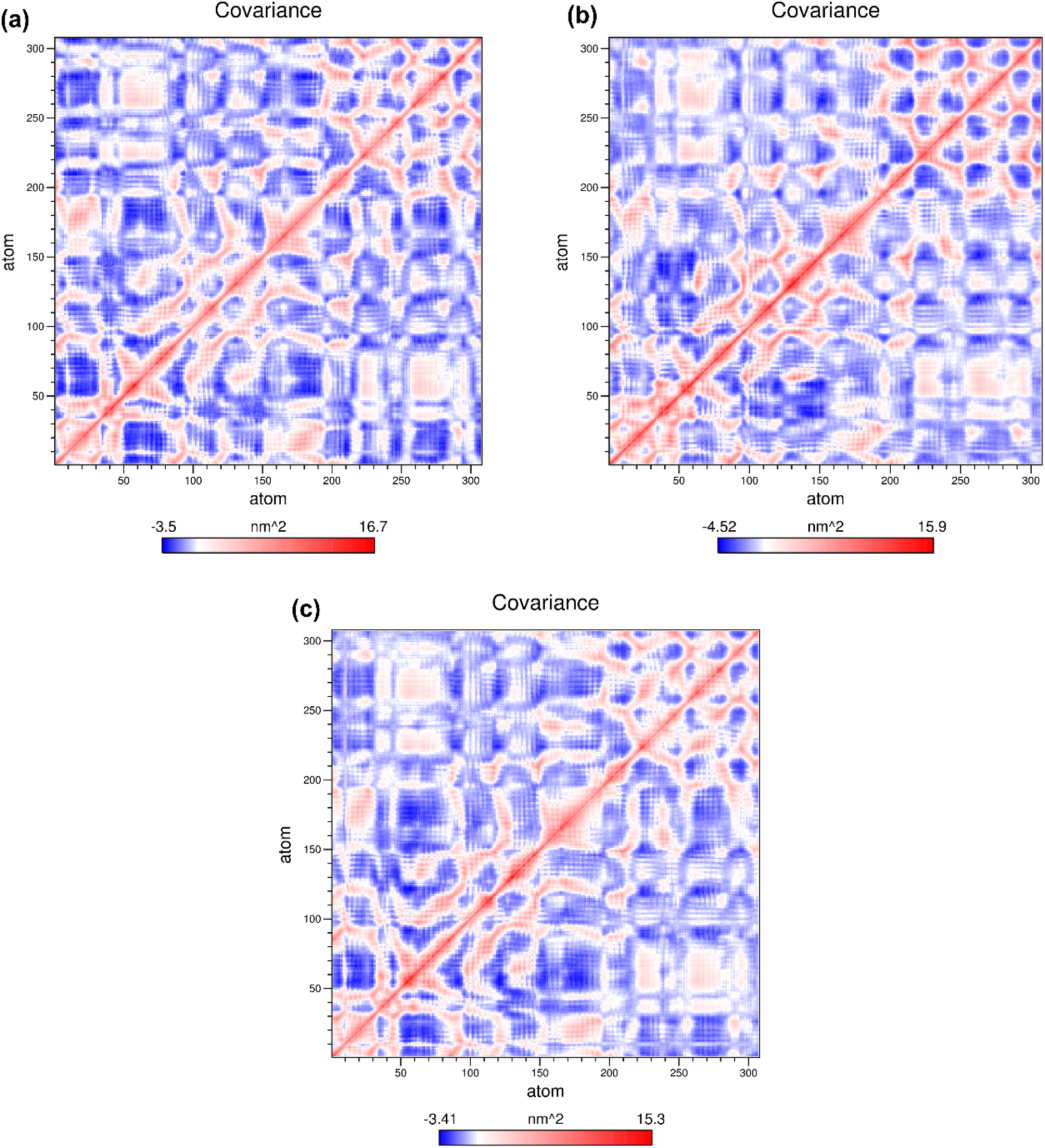
Calculation of dynamics cross-correlation matrix for the FtsZ-ligand complexes based on the Cα-residue (**a**) FTsZ-ZINC524729297, (**b**) FTsZ- ZINC000604405393, (**c**) FTsZ-berberine.

The DCCM was constructed to study the impact of inhibitor binding on the correlative movements of FtsZ using the Cα atoms obtained from the MD simulation trajectories of 500 ns. The red zone in the plot indicates a positive correlation, whereas the blue zone indicates a negative correlation. The correlation coefficient, which ranges from − 1 to 1 and is represented by red and blue in Fig. 8a–c, indicates that a positive value represents movement in the same direction, whereas a negative value signifies movement in the opposite direction. The analysis revealed significantly correlated and anti-correlated motions in the FtsZ-ZINC524729297 and FtsZ- ZINC000604405393 complexes, indicating the presence of stronger cross-correlation.

### Functional analysis of molecular properties

**Fig. 9:**
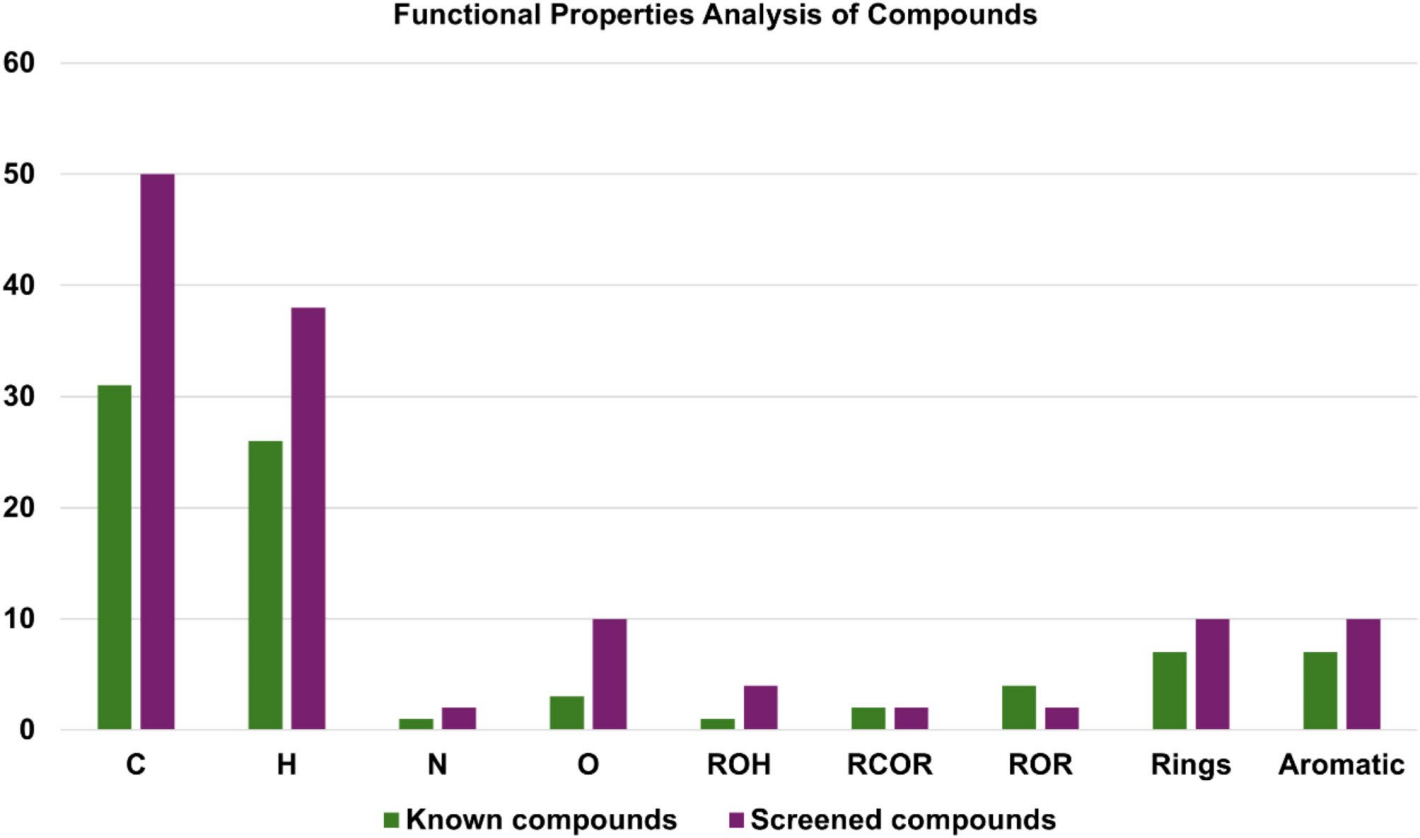
Functional analysis of molecular properties of finalized compounds and known FtsZ inhibitor.

Molecular properties of the screened compounds ZINC524729297 and ZINC000604405393, along with berberine and plumbagin. It was observed that similar numbers of atoms, functional groups, and rings were present in both anti-FtsZ compounds and the screened compounds. In the finalized screened compounds, carbon, hydrogen, nitrogen, fluorine, oxygen, and sulfur were present, along with functional groups such as secondary amines, tertiary amines, and carbonyl groups as shown in Fig. 9. Furthermore, these groups were also present in both the anti-FtsZ compounds, but the number of these groups was higher in both the screened compounds than in the known compounds. These results indicate that these screened compounds possess structural and functional characteristics associated with inhibitory activity against the *E. coli* FtsZ protein.

## Discussion

Cell division is a crucial process for the existence of all bacterial cells^27^. This process involves three steps: cell elongation, septum formation, and cell division into two identical cells. Filamentous temperature-sensitive mutant Z (FtsZ) is a crucial cytoskeletal protein involved in cell division in most bacteria. During cell division, FtsZ forms a ring-like structure known as the Z-ring at the division site and serves as a scaffold for recruiting the multiprotein complex known as the divisome^28^. The Z-ring, which is highly dynamic and consists of FtsZ along with various other proteins, also plays a crucial role in the localization and stability of divisional proteins through protein-protein interactions. The Inhibition of FtsZ can disrupt cell division, making it a promising therapeutic target^12^. Therefore, we used machine algorithms to distinguish anti-FtsZ compounds from non-inhibitory compounds. The machine learning classifiers were assessed considering several statistical metrics, including kappa statistic, sensitivity, specificity, accuracy, and ROC analysis. J48 was selected as the best classifier with highest kappa statistic value of 0.62 and high accuracy. This result indicates that the model’s predictions closely related to the actual class. The high ROC value validates the robustness of the J48 model in correctly distinguishing between the two classes. Sensitivity and specificity analysis revealed the superiority of J48 model in correctly recognizing positive instances, whereas Random Forest showed lower sensitivity. Overall, J48 was the best performing classifier, followed by LMT and Random Forest. This study involved the screening of 1072 berberine analogues based on machine learning algorithms. A set of 740 active compounds identified by J48 classifier and further screened based on their pharmacokinetic and toxicological properties. A total of 60 filtered molecules were subjected to virtual screening with FtsZ based on binding affinity to identify the best lead molecules. A set of 20 compounds with the highest binding affinity, along with berberine, were subjected to molecular docking analysis to determine their binding energies and IC. Lower binding energies and IC values are key parameters for evaluating the therapeutic potential of a drug in inhibiting enzyme activity. A lower binding energy is associated with lower IC values. The docking outcome suggested FtsZ-ZINC524729297, FtsZ-ZINC000604405393, FtsZ-ZINC000072312902, FtsZ-ZINC000085341281, and FtsZ-DB08387 complexes exhibited binding energy of − 8.73 kcal/mol, − 8.55 kcal/mol, − 8.32 kcal/mol, − 8.3 kcal/mol and − 8.2 kcal/mol respectively, which was observed to be lower than berberine with binding energy of − 6.59 kcal/mol. The effectiveness of ZINC524729297, ZINC000604405393 and ZINC000085341281 is linked to the formation of four and two H-bonds, respectively, reflecting strong interactions with multiple residues in specific binding sites, resulting in better inhibition than berberine with only one H-bond. MD simulation were used to evaluate the long-term stability and structural changes of the protein–ligand complexes^29^. In the trajectory analysis, the RMSD was calculated for the protein Cα atoms and all ligand atoms^30^. The analysis showed lower RMSD values for FtsZ-ZINC524729297, FtsZ-Ft ZINC000604405393, and FtsZ-ZINC000085341281, suggesting higher stability, whereas FtsZ-berberine and FtsZ-DB08387 showed higher RMSD values, indicating decreased stability. These findings reveal the impact of ligand binding on the structural integrity of FtsZ, providing a deeper understanding of the functional effects of these interactions. The RMSF calculation of the Cα atoms of FtsZ helps assess the flexibility of each amino acid residue; higher RMSF values indicate greater flexibility, and lower values imply greater stability^31^. The FtsZ-ZINC524729297 and FtsZ-ZINC000604405393 complexes exhibited the lowest RMSF values, indicating the highest stability, whereas the FtsZ-ZINC000072312902, FtsZ-ZINC000085341281, and FtsZ-DB08387 complexes exhibited moderate stabilization. The FtsZ-berberine complex, with higher RMSF values, showed greater flexibility. These findings suggest that ZINC524729297 and ZINC000604405393 are more effective at decreasing flexibility and potentially improving functional stability. Hydrogen bonds are essential for maintaining stability of protein structure and facilitating interactions between protein and ligands^32^. H-bond analysis revealed that the FtsZ-ZINC524729297, FtsZ-ZINC000604405393 and FtsZ- ZINC000085341281 complexes formed average H-bonds of 2.3, 1.2, and 0.17, respectively, indicating stronger binding and greater structural stabilization. The radius of gyration measures protein size and density, showing different levels of compaction in the ligand-bound state^33^. This analysis indicates that ligand binding influences the compactness of FtsZ. Lower Rg values correspond to higher protein compactness, while the higher Rg values indicate reduced protein compactness^34^. The results showed that all the ligands reduced protein compactness compared with berberine. Another parameter for measuring stability is the interaction energy, where a lower energy denotes greater stability, while a higher energy indicates less stability. The complexes like FtsZ-ZINC524729297 and FtsZ- ZINC000604405393 exhibited lower energy and stability, while other complexes showed comparatively higher energy, making these complexes unstable. SASA analysis revealed that ligand binding altered the solvent-accessible surface area of FtsZ^35^. Complexes such as FtsZ-ZINC524729297, FtsZ-ZINC000604405393 and FtsZ-ZINC000072312902 exhibited decreased SASA, indicating reduced solvent exposure, whereas FtsZ-berberine and other complexes exhibited increased SASA, indicating greater surface accessibility. These findings help understand the dynamics and solvent interactions of FtsZ. In MM-PBSA analysis, a compound’s ranking is generally determined by calculating the binding free energy, which is a sum of various energy components such as van der Waals interactions, electrostatic interactions and solvation energies. A lower binding free energy indicates higher binding affinity. This analysis showed that the FtsZ-ZINC524729297 and FtsZ-ZINC000604405393 complexes exhibited the most favourable binding energy, reflecting a stronger interaction with FtsZ and a higher binding affinity compared to the other complexes. PCA analysis was employed to evaluate the impact of inhibitor binding on the motion and structural alterations of the target protein^36^. In the 2D projection plot, the complex occupying less phase space indicates the stable cluster, while the complex occupying more phase space indicates the unstable cluster^37^. The layout revealed that the FtsZ-ZINC524729297 and FtsZ-ZINC000604405393 complexes occupied less space compared to other complexes, suggesting a more stable conformation and has the potential to bind with FtsZ. The decreased conformational dynamics may result from optimized interactions that restrict the backbone dynamics of the protein. The FEL analysis offers significant insights into the structural changes inside the FtsZ-ligand complexes, providing valuable information on their stability. The deep blue basins in the FEL plots indicate a stable conformation^38^. Complexes such as FtsZ-ZINC524729297 and FtsZ-ZINC000604405393 showed high stability, characterized by fewer transition states and distinct energy minima, whereas the FtsZ-berberine complex exhibited lower stability with multiple transition states. The DCCM analysis revealed the impact of inhibitor binding on protein motions, where positive correlations indicating coordinated motions and negative correlations indicating counter movements^39^. In this analysis, FtsZ-ZINC524729297 and FtsZ-ZINC000604405393 showed both correlated and anti-correlated motions, indicating structural integrity during the simulation. The functional group analysis revealed that the average atoms count, functional group counts, and ring counts were higher in ZINC524729297 and ZINC000604405393 than berberine and plumbagin respectively. This result indicates that the selected compounds would have inhibitory activity against FtsZ protein. Consequently, ZINC524729297 and ZINC000604405393 are ideal candidate compared to the other ligands. The results demonstrated that compound ZINC524729297 and ZINC000604405393 exhibited a higher stability profile than other ligands, highlighting the significance of inhibitor binding in maintaining FtsZ stability and understanding its therapeutic potential. Therefore, the promising therapeutic effects of ZINC524729297 and ZINC000604405393 need to be tested through in vitro and in vivo studies.

## Materials and methods

### Retrieval of target protein

The crystal structure of FtsZ from *E. coli* is available in RCSB-PDB and downloaded using PDB ID 8GZY. The structure of FtsZ was determined using X-ray crystallography with a resolution of 2.60 Å. All water molecules and heteroatoms were removed from the original structure using PyMOL software, and the 3D structure was saved in the PDB format. The overall energy minimization of the protein structure was performed using 2000 steps of the steepest descent and conjugated gradient algorithms in SPDBV, employing the vacuum force field of GROMOS 43B1^40^. Finally, the energy-minimized structure was utilized for molecular docking and simulation studies.

### Data collection and ligand preparation

In this study, anti-FtsZ compounds with their inhibitory concentration (IC50) against *E. coli* were retrieved from ChEMBL (CHEMBL3999) database. Compounds without IC50 values were excluded from this study. A total of 44 compounds for FtsZ were considered for model construction and validation. After initial model construction we calculated the pIC50 by taking negative logarithmic of IC50 with base 10. Molecules with pIC50 value more than 7 were considered as active, and less than 7 were considered as inactive. Based on a literature review, a small organic molecule, berberine, inhibited the FtsZ cell division protein^41^. For the test dataset, a total of 1072 berberine analogues were retrieved from the web-based tool SwissSimilarity under ChEMBL approved drug, Drugbank and Commercial ZINC drug like categories for evaluation^42^. Furthermore, the 3D structure of all the compounds were available and processed for further evaluation.

### Descriptor calculation and data set preprocessing

Chemical descriptors are characteristic features of small molecules that determine their activity. All the compounds were saved in 3D-SDF format using O’Bable software and exported to PaDel program to calculate the descriptors for all the compounds. A total of 2757 descriptors including fingerprints and one-dimensional, two-dimensional and three-dimensional characteristics were obtained. The complete dataset was then screened using Waikato Environment for Knowledge Analysis (WEKA) software to generate predictive models for machine learning^43^. For feature selection process, several numbers of functions in WEKA were considered like correlation, attribution, evaluation and replacement of missing values. A valid model was built using ten-fold CV approach by employing three different classifiers, namely, random forest, LMT and J48 for the training dataset. In a 10-fold CV, the entire training data is divided into ten distinct subsets or folds. The performance of predictive model using the combined data from the remaining nine folds underwent evaluation for each fold, resulting in ten different performance metrices^44^. The performance of each classifier’s model was evaluated using confusion matrix and evaluation statistics, including specificity, sensitivity, prediction accuracy and Kappa statistic. The following formulas were applied to evaluate the accuracy of the models.

$$\text{Binary classification accuracy} = \:\frac{\text{T}\text{N}\:+\:\text{T}\text{P}}{(\text{T}\text{N}\:+\:\text{T}\text{P}\:+\:\text{F}\text{N}\:+\:\text{F}\text{P})}$$

$$\text{Sensitivity} =\:\frac{\text{T}\text{P}}{(\text{F}\text{N}\:+\:\text{T}\text{P})}$$

$$\text{Specificity} = \:\frac{\text{T}\text{N}}{(\text{F}\text{N}\:+\:\text{T}\text{N})}$$

$$\text{Kappa statistics} = \:\frac{2\:\times\:(\text{T}\text{P}\:\times\:\:\text{T}\text{N}\:-\text{F}\text{N}\:\times\:\:\text{F}\text{P})}{(\text{F}\text{P}\:+\:\text{T}\text{P})(\text{F}\text{P}\:+\:\text{T}\text{N})\:+(\text{F}\text{N}\:+\:\text{T}\text{P})(\text{F}\text{N}\:+\:\text{T}\text{N})}$$

### Ligand based virtual screening of potential compounds and physicochemical properties analysis

Virtual screening is a crucial technique for identifying new potent compounds capable of interacting with specific target proteins to regulate their activity. The analogues were screened for desirable adsorption, distribution, metabolism, and excretion (ADME) properties using Swiss ADME software. The criteria for the selection of analogues were as follows: molecular weight $\:\le\:$ 500 Da, number of hydrogen bond donors $\:\le\:\:$5, hydrogen bond acceptors $\:\le\:\:$10, log *P* $\:\le\:\:$5, rotatable hydrogen bonds $\:\le\:\:$10 and no violations in any rules with lower synthetic accessibility. The topological Polar Surface Area (TPSA) is used to measure the bioavailability of a drug molecule. TPSA is closely linked with the hydrogen bonding potential of a compound and TPSA value less than 140 Å^2^ indicates good intestinal absorption^45,46^. Other criteria, such as lead likeliness and synthetic accessibility, were also considered to filter the analogues. The toxicity of the filtered ligands was predicted using ProTox 3.0^47^. These compounds were checked for cardiotoxicity, hepatotoxicity, mutagenicity, and carcinogenicity, and only non-toxic compounds with an LD50 $\:\ge\:$ 1000 mg/kg were selected^40,48^. Autodock vina (PyRx) was used for virtual screening of non-toxic ligands against the FtsZ protein^49^. Finally, the ligands with the lowest binding energies were selected for further analysis.

### Molecular docking studies

The screened molecules were then subjected to molecular docking analysis with the FtsZ protein using Autodock tools 4.2.6 to understand the intermolecular interactions^50^. The selected compounds were optimized to obtain a 3D structure for molecular docking. The protein was stabilized by adding polar hydrogen atoms along with Kollman charges and merging non-polar hydrogen atoms. The ligand molecules remained in a flexible conformation, whereas the protein was maintained in a rigid position. This flexibility is important for the ligand as it allows the adoption of multiple orientations and conformations during the docking process, which can influence binding interactions. Gasteiger charges were added to optimize the protein. In the molecular docking grid center is the central point in 3D space that defines the area around which a ligand is allowed to explore potential binding orientation within a protein’s active site^50^. The active site coordinates were taken based on literature review and x = 0.193, y = − 35.688 and z = 23.784 centered in a grid box with a dimensions of 60 × 60 × 60 Å^3^^52^. This active site was defined in inter-domain clefts based on the literature review. The Lamarckian genetic algorithm was used to explore the various possible conformations of the target protein and ligand and predict the binding interaction between the protein and ligands^52^. Two different programs, Autogrid4 and Autodock4, were used to generate docked complexes of the target protein and ligands. Autodock is the main software, while Autogrid calculates the non-covalent interaction energy generating an electrostatic potential grid map. An important feature of Autodock4 is the ability to model receptor flexibility by shifting the side chains^50^. Docked complexes with the lowest binding energy were generated using Lamarckian and genetic algorithms^53,54^. The 3D and 2D conformers were visualized using the UCSF ChimeraX and Discovery Studio Visualizer, respectively^55^.

### Molecular dynamics (MD) simulation

The docked complexes with high binding affinities and low binding energies were subjected to molecular dynamics simulation analysis. The stability of the protein-ligand complexes was analyzed for a duration of 500 ns using the GROMACS version 2024.2 package^56^. The ligand and protein topologies were prepared using the CGenFF server and the CHARMM36 all-atom field, respectively^57^. The complexes were placed in a dodecahedron box, maintaining a uniform edge distance of 1.2 nm. The system was placed in a solvent environment using a simple point-charge (TIP3P) water model and neutralized with two counter ions, Na^+^ and Cl ^−^. The energy minimization of the system was carried out using the steepest descent algorithm (50,000 steps) with a convergence tolerance of 1000 kJ/mol/nm. The equilibration was a two-step process, which involved the NVT (constant volume) ensemble with a leap-frog integrator and the NPT (constant pressure and temperature) ensemble with the Parrinello–Rahman barostat to stabilize the pressure at 1 bar and temperature at 300 K, respectively. The electrostatic interactions were treated using the particle-mesh Ewald algorithm. Finally, the MD simulation was performed for 500 ns with a 2 fs time step, followed by analyses including RMSD, RMSF, radius of gyration, interaction energy, SASA, and hydrogen bond trajectories, which were subsequently visualized^48,58^.

### MM-PBSA analysis

The Molecular Mechanics Poisson-Boltzmann Surface Area (MMPBSA) is a computational analysis helps to integrate high-throughput MD simulation with the estimation of binding free energies (ΔG) of the protein-ligand interactions^59^. The analysis provided various energy calculations, including VDWAALS, EEL, EGB, ESURF, GGAS, GSOLV^37^. We used gmxMMPBSA to calculate MM-PBSA for protein-ligand complexes in the GROMACS package^60^. The MM-PBSA based binding free energy was calculated by removing the periodic boundaries and water molecules of FtsZ with the ligands (ZINC ID). The molecular geometry of the protein-ligand complex was optimized by energy minimization using molecular mechanics calculations. The ΔG_bind_ values of the complexes were calculated using the following equation:

$$\:\varDelta\:G_{binding} = \:\varDelta\:G_{complex} - (\:\varDelta\:G_{protein} + \:\varDelta\:G_{ligand})$$

Here, ΔG_binding_ denotes the free energy of binding and the energies of the complex, protein, and ligand are represented by ΔG_complex_, ΔG_protein,_ and ΔG_ligand,_ respectively. The overall binding energy for each complex is determined by various components, including van der Waals force, electrostatic energy, polar solvation energy using Poisson–Boltzmann methods, non-polar solvation energy determined through Poisson–Boltzmann methods, gas-phase molecular mechanics free energy, and solvation free energy^61,62^.

### Principal component analysis (PCA)

The principal component was calculated through the diagonalization and solvation of the eigenvalues and eigenvectors of the covariance matrix. The magnitude of motion is represented by eigenvalues, whereas the direction of motion is indicated by eigenvectors^63^. Alterations in the C-alpha atoms for each residue are included in the covariance matrix, which produces orthogonal eigenvectors along with their corresponding eigenvalues. Principal component analysis (PCA) of all protein-ligand complexes was performed using the GROMACS analysis tool. The covariance matrix was diagonalized and calculated using the GROMACS software package, gmx covar. The overlap between the principal components and trajectory coordinates was calculated using gmxanaeig, another GROMACS tool^64,65^.

### Free energy landscape (FEL)

FEL depicts the possible conformation a protein can adopt during MD simulations, along with the corresponding Gibbs free energy. The FEL depicts two variables that show specific system properties and determine the conformational variability^63^. The FEL was calculated based on the probability distribution of the essential plane formed by the first two eigenvectors. Construction of the FEL was performed using the gmx sham script, utilizing the PCA values obtained^66^.

### Dynamic cross-correlation matrix (DCCM) analysis

The DCCM analysis was performed to calculate the temporal shifts of Cα atoms in the protein resulting from the binding of the ligand to the protein. The correlation matrix was constructed for all the Cα atoms, which facilitated an in-depth examination of the domain relationships^67^.

### Functional properties analysis

The functional analysis of the molecular properties of the compounds was performed by R v 4.4.2 software using ChemmineR library. The molecular properties of the lead compounds, including functional group counts of carbonyl, nitrile, primary amine, secondary amine, tertiary amines, ester group, carboxyl group, hydroxyl group, ether group, and rings, were analyzed and compared with those of known FtsZ inhibitors berberine and plumbagin^68^.

## Conclusion

The FtsZ protein in *E. coli* provides important insights into its role in cell division. Inhibition of this cell wall synthesis protein can lead to a new direction for developing drugs and enhancing the therapeutic regimen to treat *E. coli* infections. In the present study, virtual screening of berberine analogues using the machine learning algorithm revealed that ZINC524729297 and ZINC000604405393 are ideal FtsZ inhibitor. We conducted various in silico experiments including ADME analysis, toxicity analysis, molecular docking, MD simulation, MM-PBSA binding free energy calculation, PCA, FEL, and DCCM analysis. The results show that the complex exhibit strong binding affinity and low binding energy, indicating better inhibition against FtsZ, surpassing the reference drug berberine. MD simulation and MM-PBSA calculations confirmed the stability of ZINC524729297 and ZINC000604405393 as potential FtsZ inhibitor. The present in silico study encourages the experimental validation of ZINC524729297 and ZINC000604405393 to confirm its therapeutic effectiveness as an FtsZ inhibitor.

## Electronic supplementary material

Below is the link to the electronic supplementary material.

Supplementary Material 1

Supplementary Material 2

Supplementary Material 3

Supplementary Material 4

Supplementary Material 5

Supplementary Material 6

## Data Availability

All data generated during this study are included in this article as tables and figures.The structure analysed in this current study were taken from PDB repository [PDB ID: 8GZY] (https://www.rcsb.org/). The raw data generated and analysed in this study represented in Supplementary Information [SI].
