# Supplementary material for: Unveiling Berberine analogues as potential inhibitors of Escherichia coli FtsZ through machine learning molecular docking and molecular dynamics approach
Source: Sci Rep. 2025 Apr 26;15:14668. doi: 10.1038/s41598-025-98835-x (PMC12033256; doi:10.1038/s41598-025-98835-x)
Supplement: Supplementary file 6 — Supplementary Material 6 [file 41598_2025_98835_MOESM6_ESM.docx]

**Supplementary Table Legends**

**Table 1.** List of active compounds screened by machine learning algorithm

**Table 2.** Pharmacokinetic properties of berberine analogues

**Table 3.** Toxicity profile of 60 compounds

**Table 4.** Binding affinity of different ligands with FtsZ

**Table 5.** Binding Energy of FtsZ with different ligands during docking


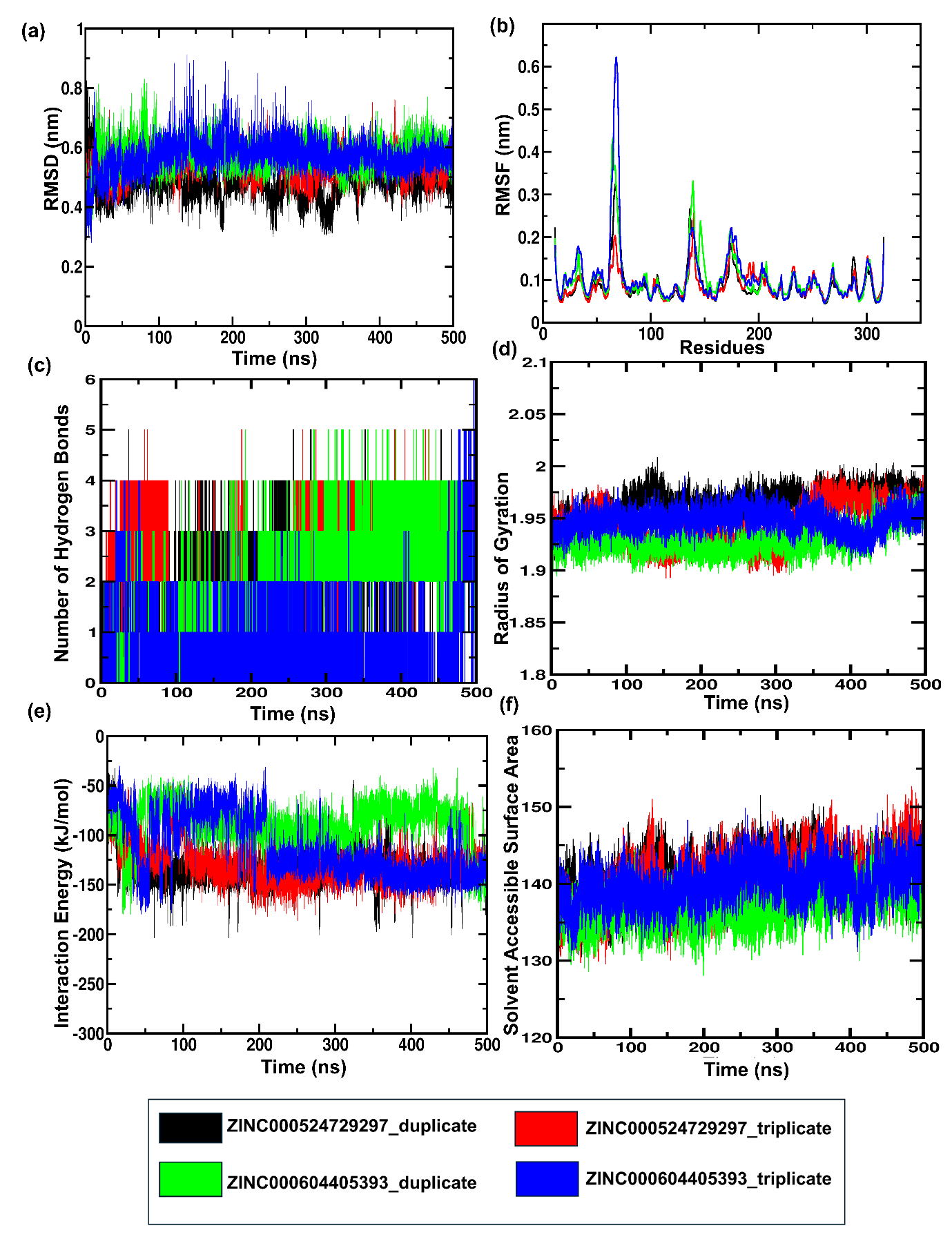


**Fig. 1** Molecular dynamics simulation analysis of protein-ligand **(a)** Root means square deviation (RMSD), **(b)** Root mean square fluctuation (RMSF) associated with the number of residue, **(c)** Intermolecular H-bond, **(d)** Radius of gyration, **(e)** Interaction energy, **(f)** Solvent Accessible Surface Area (SASA)


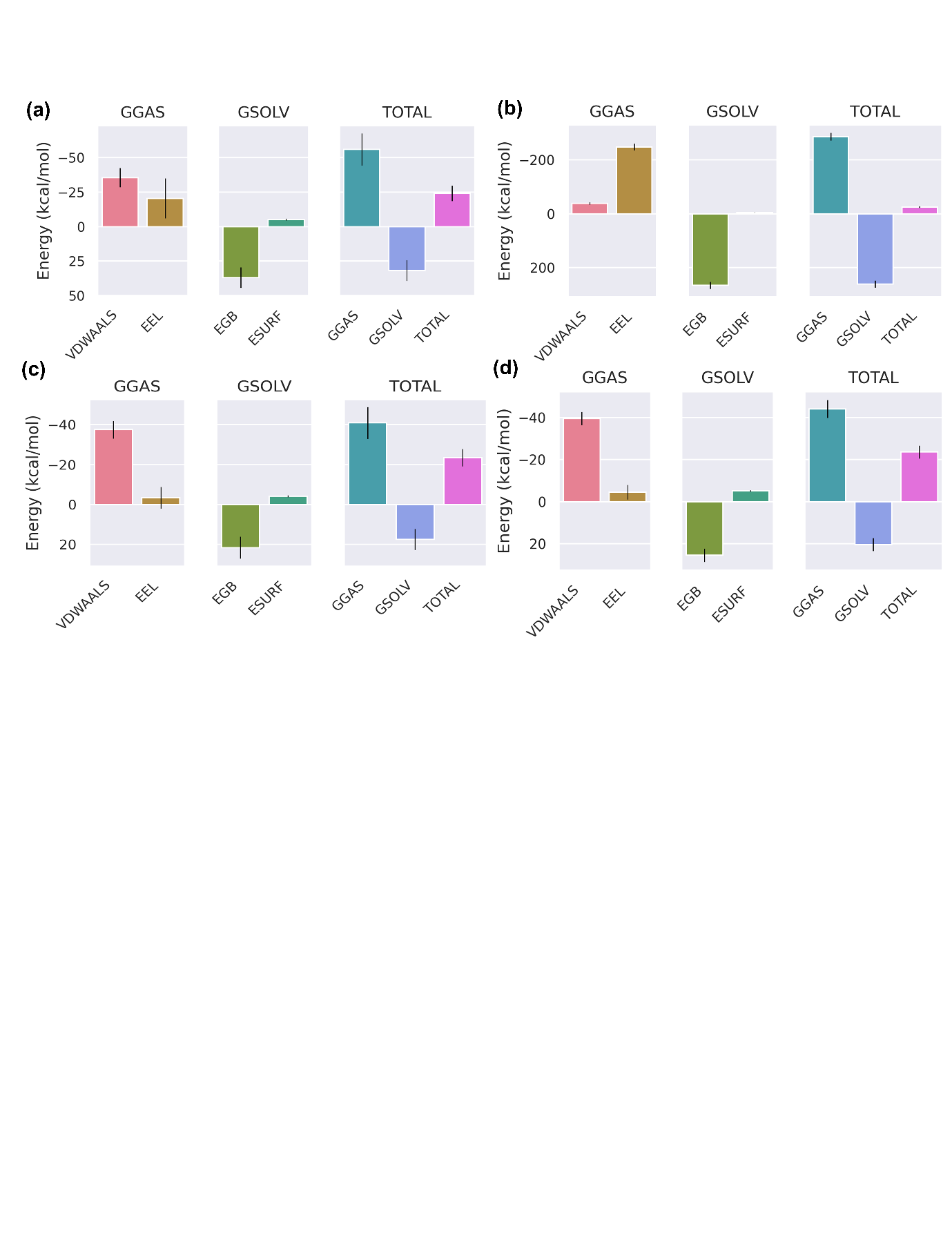


**Fig. 2** MM-PBSA analysis of individual residues contributing to total binding free energy **(a)** FtsZ-ZINC524729297_duplicate, **(b)** FtsZ- ZINC524729297_triplicate, **(c)** FtsZ- ZINC000604405393_duplicate, **(d)** FtsZ- ZINC000604405393_triplicate


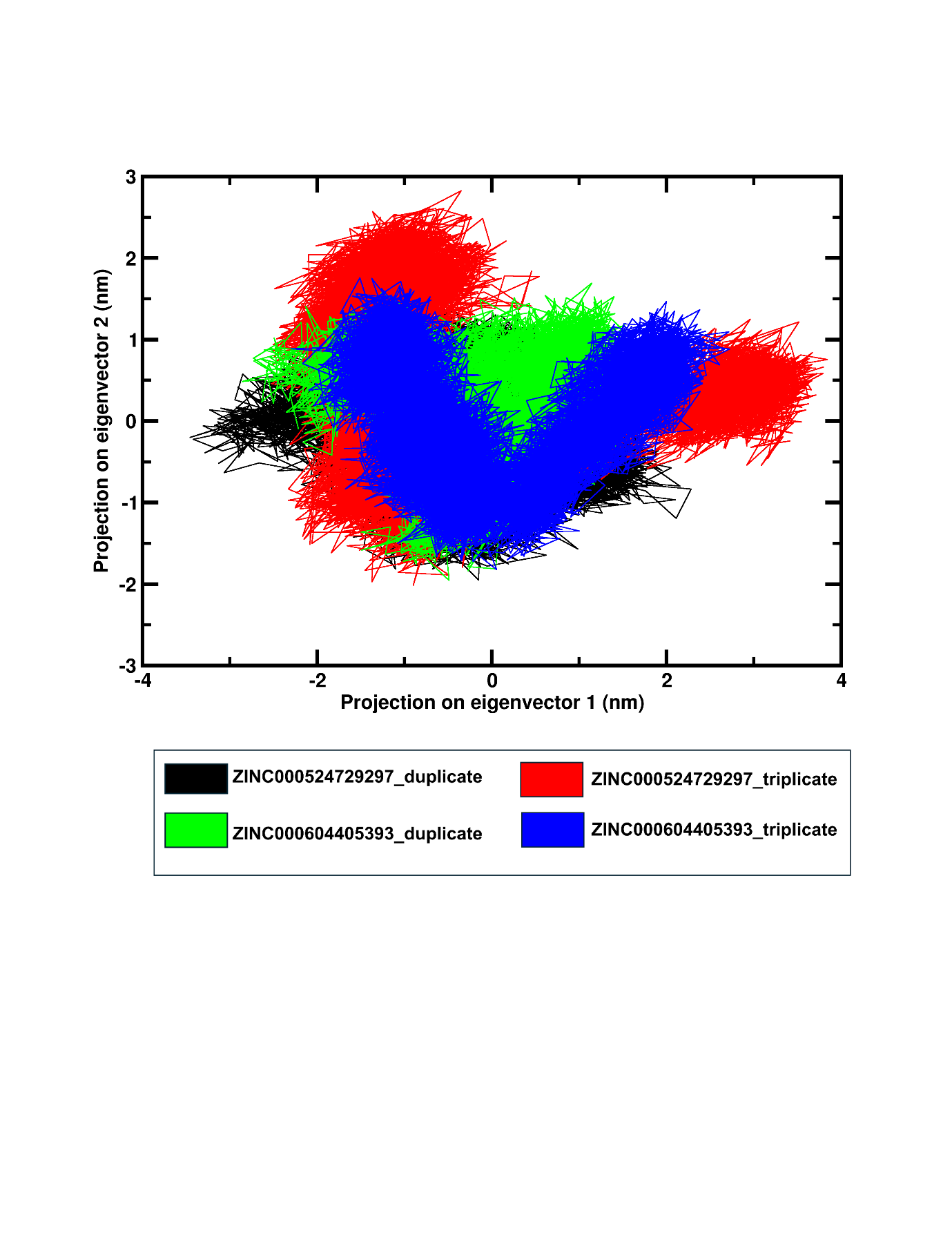


**Fig. 3** The 2D conformational projection of Principal Component Analysis (PCA) of FtsZ-ZINC524729297_duplicate, FtsZ-ZINC524729297_triplicate, FtsZ-ZINC000604405393_duplicate, FtsZ-ZINC000604405393_triplicate


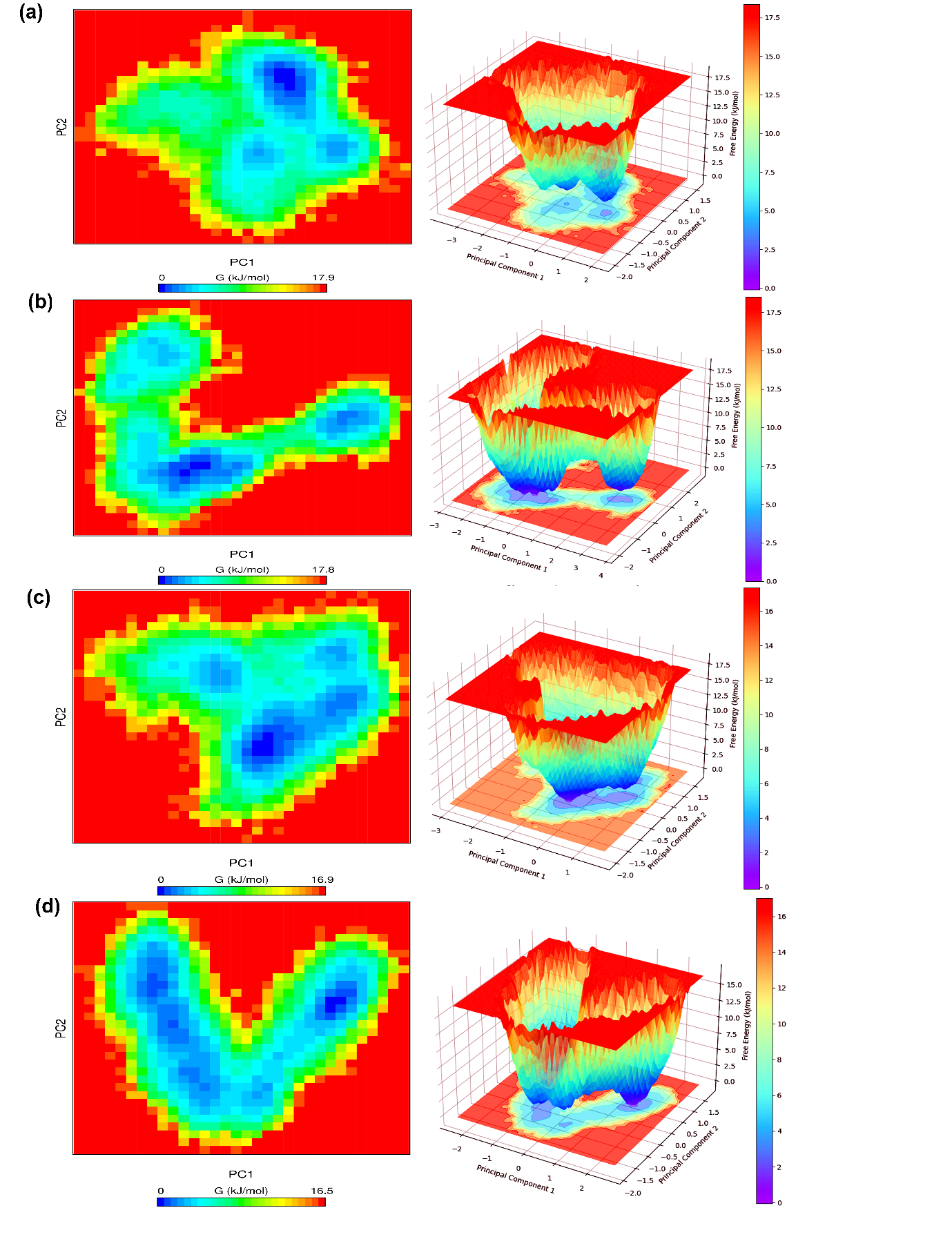


**Fig. 4** The graphical depiction of PCA based Free Energy Landscape (FEL) analysis of FtsZ-ligand complexes **(a)** FtsZ-ZINC524729297_duplicate, **(b)** FtsZ-ZINC524729297_triplicate, **(c)** FtsZ-ZINC000604405393_duplicate, **(d)** FtsZ- ZINC000604405393_triplicate
